# Designing a Novel Multi-Epitope Trivalent Vaccine Against NDV, AIV and FAdV-4 Based on Immunoinformatics Approaches

**DOI:** 10.3390/microorganisms13122744

**Published:** 2025-12-02

**Authors:** Jiashuang Ji, Xiaofeng Dong, Xiangyi Liu, Mengchun Ding, Yating Lin, Yunhang Zhang, Wuchao Zhang, Baishi Lei, Wanzhe Yuan, Kuan Zhao

**Affiliations:** 1 College of Veterinary Medicine, Hebei Agricultural University, Baoding 071001, China; jjs0820@126.com (J.J.); dongxiaofeng0519@126.com (X.D.); 15028578986@163.com (X.L.); 13231377179@163.com (M.D.); a664825313@163.com (Y.L.); zhangyunhang@hebau.edu.cn (Y.Z.); zhangwuchao168@126.com (W.Z.); leibaishi2000@163.com (B.L.); yuanwanzhe@126.com (W.Y.); 2 Veterinary Biological Technology Innovation Centre of Hebei Province, Hebei Agricultural University, Baoding 071000, China

**Keywords:** genotype VII NDV, H9N2, FAdV-4, epitope vaccine, immunoinformatics

## Abstract

The diseases caused by genotype VII Newcastle disease virus (NDV), H9N2 avian influenza virus (AIV), and fowl adenovirus serotype 4 (FAdV-4) continue to threaten the global poultry industry. However, no broad-spectrum vaccines provide simultaneous protection against these three pathogens. This study employed bioinformatics and immunoinformatics approaches to design a multi-epitope vaccine, named NFAF, which consists of B-cell, cytotoxic T lymphocyte (CTL) epitopes, and helper T lymphocyte (HTL) epitopes derived from hemagglutinin-neuraminidase (HN) and fusion (F) proteins of genotype VII NDV, hemagglutinin (HA) protein of H9N2, and Fiber2 protein of FAdV-4. The vaccine candidate was predicted to have non-allergenic properties, non-toxicity, high antigenicity, and favorable solubility. Each of its constituent antigenic epitopes has a high degree of conservation. Molecular docking demonstrated stable binding between NFAF and chicken Toll-like receptor (TLRs) and major histocompatibility complex (MHC) molecules. NFAF was expressed in soluble form in *Escherichia coli* and purified. Polyclonal antibodies against all three target viruses showed specific binding to NFAF. In vitro experiments revealed that NFAF effectively stimulated chicken peripheral blood mononuclear cells (PBMCs) and induced Th1, Th2, and pro-inflammatory cytokine production, confirming its immunogenicity, and increased the mRNA expression of the key signaling molecules *MyD88* and *NF-κB*. These results suggested that NFAF could therefore be an efficacious multi-epitope vaccine against genotype VII NDV, H9N2, and FAdV-4 infections.

## 1. Introduction

Newcastle disease (ND), avian influenza (AI), and Hepatitis-Hydropericardium Syndrome (HHS) are three major viral infectious diseases that pose serious threats to the global poultry industry and have resulted in substantial economic losses.

NDV, which causes ND, is an enveloped, single-stranded, negative-sense RNA virus and genetically classified into two classes, I and II. Class II shows considerable diversity and is divided into more than 20 genotypes [1]. Genotype VII viruses under Class II are now the most widespread in chickens and waterfowl across the globe. NDV possesses a genome composed of six major structural genes that sequentially encode Nucleoprotein (NP), Phosphoprotein (P), Matrix protein (M), F, HN, and Large polymerase protein (L) [2]. During viral infection, HN and F are especially important. F enables fusion between the viral envelope and the host cell membrane. HN plays pivotal roles in receptor binding, promotion of neuraminidase activity, and facilitation of membrane fusion [3,4]. Given their essential functions and their proven capacity to elicit potent neutralizing antibodies, F and HN represent prime targets for genetically engineered subunit vaccines. This makes them central to efforts in designing new vaccines.

AIV belongs to the Orthomyxoviridae family and is classified within the Influenzavirus A genus [5]. Subtypes of AIV are defined by variations in their surface glycoproteins, HA and neuraminidase (NA) [6]. Among these, H9N2 is considered a low pathogenic avian influenza virus (LPAIV) and has become highly prevalent in China and across the globe [7]. When H9N2 co-infects with other pathogens, it can exacerbate the disease, causing severe respiratory signs, stunted growth, reduced egg production, and immunosuppression in poultry [8,9]. Therefore, vaccine development against this virus is urgently needed. HA protein plays a central role in viral infection and host immunity and is thus a major focus of vaccine research. It consists of two subunits, HA1 and HA2 [10]. The HA1 subunit binds to sialic acid receptors on host cells, whereas the HA2 subunit is responsible for mediating membrane fusion [11]. Due to its ability to elicit neutralizing antibodies, HA is often selected as the target antigen in AIV subunit vaccine development [12].

HHS caused by FAdV-4 was characterized by the presence of pericardial effusion and inclusion body hepatitis [13,14]. FAdV-4 belongs to the genus Aviadenovirus within the family Adenoviridae. It is a non-enveloped virus with a double-stranded DNA genome and a diameter of 70 to 90 nm [15]. The viral capsid is assembled from major structural proteins including Hexon, Penton, and Fiber. Fiber protein is further differentiated into Fiber1 and Fiber2 [16]. Among these, Fiber1 contributes critically to viral replication and assembly, while Fiber2 mediates viral entry by interacting with host nuclear transport proteins (KPNA3/4) through its N-terminal domain [17]. Notably, Fiber2 induces high-titer neutralizing antibodies and promotes CD4^+^ T cell proliferation, highlighting its enhanced immunoprotective properties [18]. Consequently, Fiber2 is currently considered the preferred target antigen for the development of FAdV-4 subunit vaccines.

The development of efficient and safe vaccines is essential for controlling infections caused by genotype VII NDV, H9N2, and FAdV-4. Advances in bioinformatics and immunoinformatics have opened new avenues for rational vaccine design [19]. These approaches enable the in silico prediction and selection of antigenic epitopes, including CTL epitopes, HTL epitopes, and B-cell epitopes [20]. These epitopes can then be concatenated to construct multi-epitope vaccine candidates. Immunoinformatics tools facilitate the assessment of key immunological characteristics, such as antigenicity, allergenicity, and toxicity. The resulting multi-epitope vaccines are capable of stimulating CD8^+^ and CD4^+^ T lymphocytes as well as activating B lymphocytes, promoting comprehensive cellular and humoral immunity [21]. These approaches offer significant advantages over traditional methods, including decreasing the time and cost of vaccine development, and allowing for the study of non-cultivable or high-risk microorganisms. Bioinformatics and immunoinformatics approaches have been successfully employed to design multi-epitope vaccines against goatpox virus [22], hepatitis C virus [23], Ebola virus [24], and severe acute respiratory syndrome coronavirus 2 [25]. Nevertheless, a multi-epitope vaccine simultaneously targeting genotype VII NDV, H9N2, and FAdV-4 remains unreported.

In this study, we designed and constructed a multi-epitope vaccine targeting genotype VII NDV, H9N2, and FAdV-4 using immunoinformatics strategies, followed by comprehensive bioinformatics analysis. Furthermore, we validated its antigenicity and immunogenicity through a series of in vitro experiments. This study represents the first report of a multi-epitope vaccine designed to confer broad protection against genotype VII NDV, H9N2, and FAdV-4. It establishes a robust theoretical and experimental basis for the development of a combined vaccine aimed at the effective prevention and control of these pathogens.

## 2. Materials and Methods

### 2.1. Protein Sequence Retrieval

The entire amino acid sequences of HN and F proteins of genotype VII NDV (MK342603.1), HA protein of H9N2 (MW097976.1) and Fiber2 protein of FAdV-4 (KY636400.1) in FASTA format were retrieved from the National Center for Biotechnology Information (NCBI) (https://www.ncbi.Nlm.nih.gov/, accessed on 30 November 2024) database for comprehensive epitope mapping and rational design of a chimeric multi-epitope vaccine.

### 2.2. Prediction of Linear B-Cell Epitopes

B-cell epitopes serve as essential building blocks in the development of multi-epitope vaccines, as they play a central role in humoral immunity through the induction of specific neutralizing antibodies that protect the host against pathogen invasion [26]. To identify immunodominant linear B-cell epitopes, ABCpred (https://webs.iiitd.edu.in/raghava/abcpred/, accessed on 30 November 2024) was employed owing to its high predictive accuracy. A prediction threshold of 0.85 was established, and the antigenicity index was concurrently applied to evaluate the antigenic potential of the predicted epitopes.

### 2.3. Prediction of CTL Epitopes

CTLs recognize viral peptides presented by MHC I molecules, enabling direct killing of infected cells and viral clearance, which constitutes a vital defense mechanism in cellular immunity [27]. The binding epitopes of MHC I were predicted by employing NetMHCcons 1.1 (https://services.healthtech.dtu.dk/services/NetMHCcons-1.1/, accessed on 30 November 2024) in the IEDB, with the length specified as 9 amino acids. Previous studies have indicated that the chicken B-F alleles are comparable to human class I alleles in antigen presentation possess the capacity to induce immune responses [28]. Thus, the most appropriate human alleles for chickens were selected from prior research. For MHC I, the alleles HLA-B*40:06, HLA-B*41:04, and HLA-B*41:03 were chosen. Regarding the determination of binding strength, a threshold of IC50 < 500 nM was defined for strong—binding epitopes, with their percentage rank designated as 0.5. An IC50 > 500 nM was set as the criterion for weak-binding epitopes, and their percentage rank was set at 2.

### 2.4. Prediction of HTL Epitopes

HTLs are pivotal in initiating the pathways of B cells and CTLs. This function is fundamental in offering essential support for both humoral and cellular immunity [29]. The MHC II binding epitopes were predicted using the NetMHCIIpan 4.3 (https://services.healthtech.dtu.dk/services/NetMHCIIpan-4.3/, accessed on 30 November 2024) within the IEDB, with an epitope length of 15 amino acids. A percentile rank threshold of 0.5 was applied to define strong-binding epitopes, while a threshold of 2 was used for weak-binding epitopes. Candidate HTL epitopes were selected based on these defined percentile ranks. The selected alleles included DRB1*1482, DRB1*1366, DRB1*1310, and DRB1*1445 [28].

### 2.5. Multiple Epitope Antigens Design

The hydrophilicity index of the candidate epitopes was calculated using the Expasy ProtParam tool (https://web.expasy.org/protparam/, accessed on 8 December 2024). Priority was given to epitopes that simultaneously encompass CTL/HTL epitopes and linear B-cell epitopes, and have been previously characterized as immunodominant based on experimental studies. These selected epitopes were linked via a GPGPGLRMKLPKS linker to form a multi-epitope vaccine construct designated NFAF, with hydrophilicity gradually increasing from the core toward the outer regions. NFAF sequence was subsequently submitted to AllerTOPv2.0 (https://ddg-pharmfac.net/allertop_test/, accessed on 12 December 2024) to predict the allergenic potential of the fusion antigen. Toxicity was assessed using Toxin Pred 2 server (https://webs.iiitd.edu.in/raghava/toxinpred2/, accessed on 12 December 2024). Antigenicity was predicted by ANTIGENpro (https://scratch.proteomics.ics.uci.edu/, accessed on 12 March 2025), while the solubility of the construct in the *E. coli* expression system was predicted using SOLpro (https://scratch.proteomics.ics.uci.edu/explanation.html, accessed on 12 December 2024). A total of 120 protein sequences each for HN and F proteins of genotype VII NDV, HA protein of H9N2, and Fiber2 protein of FAdV-4 were retrieved from NCBI (Table 1). The MEGA X software was employed to perform multiple sequence alignment of the amino acid sequences, enabling the assessment of conservation levels among the candidate epitopes within these proteins.

NFAF sequence was submitted to PSIPRED (http://bioinf.cs.ucl.ac.uk/psipred/, accessed on 19 December 2024) for prediction of its secondary structure [6]. Subsequently, the tertiary structure of the antigen was modeled using I-TASSER (https://zhanggroup.org/I-TASSER/, accessed on 19 December 2024) [30,31]. The resulting structural model was further refined by uploading it to the GalaxyRefine server (https://galaxy.seoklab.org/, accessed on 21 December 2024), where the model with the highest C-score was selected to obtain an optimized tertiary structure for downstream analysis [32].

### 2.6. Molecular Docking

To determine whether NFAF can effectively interact with immune receptors, molecular docking simulations were conducted using GRAMM (https://gramm.compbio.ku.edu/, accessed on 26 December 2024) to predict the binding between NFAF and key components of the chicken immune system, including TLR2, TLR4, MHC I, and MHC II. The docking results were visualized using PyMOL 3.0, while the specific interacting amino acid residues were analyzed and visualized through PDBsum (https://www.ebi.ac.uk/thornton-srv/databases/pdbsum/index.html, accessed on 28 December 2024).

### 2.7. NFAF Expression and Purification

According to the designed sequence, the recombinant plasmid pCold II-NFAF was synthesized and constructed by Sangon Biotech (Shanghai) Co. (Shanghai, China), and then transformed into *E. coli* BL21(DE3) competent cells to generate the recombinant strain NFAF. Protein expression was induced at 18 °C and 160 rpm with a final IPTG concentration of 0.3 M for 18 h. NFAF expression was analyzed using SDS-PAGE electrophoresis, the target protein was subsequently purified using Ni-agarose resin (Kangwei, Beijing, China), and validated through Western blot.

### 2.8. Immunoreactivity Detection

The binding capacity of the purified NFAF protein to three rabbit-derived polyclonal antibodies targeting genotype VII NDV, H9N2, and FAdV-4 was assessed using Western blot and enzyme-linked immunosorbent assay (ELISA). NFAF was mixed with 4× loading buffer (Solarbio, Beijing, China) and subjected to SDS-PAGE on a 10% polyacrylamide gel, followed by transfer onto a polyvinylidene fluoride (PVDF) membrane. The membrane was blocked at room temperature for 1 h with 5% skimmed milk diluted in Tris-buffered saline containing 0.05% Tween-20 (TBST). Subsequently, the membrane was incubated overnight at 4 °C with rabbit-derived polyclonal antibodies against genotype VII NDV, H9N2, and FAdV-4 (produced and preserved in our laboratory), each diluted at a ratio of 1:200. Following three washes with TBST, the membrane was incubated with a 1:5000 dilution of HRP-labeled goat anti-rabbit IgG secondary antibody at room temperature for 1 h. After an additional three washes, the membrane was developed using a chemiluminescent detection system.

The concentration of purified NFAF was determined using the BCA Protein Assay Kit (Solarbio, Beijing, China). The sample was subsequently diluted to 10 µg/mL with PBS and added to the microplate at 100 µL per well. The plate was incubated at 37 °C for 2 h with 100 µL/well of PBST containing 5% FBS to block non-specific binding. After blocking, serially diluted rabbit-derived polyclonal antibodies against genotype VII NDV, H9N2, and FAdV-4 were added to the respective wells, followed by incubation at 37 °C for 1 h. The plate was then washed three times with PBST. Subsequently, HRP-labeled goat anti-rabbit IgG antibody (Solarbio, Beijing, China) was added and incubated at 37 °C for 1 h, followed by three additional washes with PBST. Antibody binding was detected by the addition of TMB substrate solution (Solarbio, Beijing, China), and the plate was incubated at room temperature in the dark for 10 min. The reaction was terminated using 2 M H_2_SO_4_, and the absorbance was measured at 450 nm.

### 2.9. Isolation of PBMC from Chickens

The density gradient centrifugation method was employed to isolate mononuclear cells from chicken peripheral blood. Blood was collected from the hearts of healthy chicks into sterile heparin sodium anticoagulant tubes and gently mixed with sterile PBS at a 1:1 ratio. Five milliliters of the diluted blood was carefully layered along the tube wall into a 15 mL sterile centrifuge tube containing 5 mL of lymphocyte separation medium (Solarbio, Beijing, China). The sample was centrifuged at 500× *g* for 20 min at room temperature. Following centrifugation, distinct layering was observed. The second layer containing the white buffy coat was carefully transferred to a new 15 mL sterile centrifuge tube and washed twice with sterile PBS by centrifugation at 250× *g* for 10 min per wash.

### 2.10. Effects of NFAF on Cytokine mRNA Expression Profiles in Chicken PBMC

Chicken PBMC was cultured in a 6-well plate at a volume of 2 mL/well. Various concentrations of NFAF (10, 20, 40, and 80 µg/mL) and LPS (2 µg/mL) were added to the wells, and the cells were then incubated at 37 °C for a duration of 6 h. Total RNA was isolated from the harvested cells using TRIzol (Takara, Beijing, China). Complementary DNA (cDNA) was synthesized from the extracted RNA using HiScript III RT SuperMix (Us Everbright, Shanghai, China), which served as the template for subsequent Quantitative Real-time PCR (qPCR) analysis. The primers targeting cytokines, listed in Table 2, were obtained from NCBI and validated for their specificity and suitability in this study. The qPCR reactions were carried out using Universal SYBR Green qPCR Supermix (Us Everbright, Shanghai, China), following the manufacturer’s recommended protocol. Amplification was performed on a QuantStudio^TM^ 5 Real-Time PCR System (Thermo Fisher, Shanghai, China) with the following cycling conditions: initial denaturation at 94 °C for 30 s, followed by 40 cycles of denaturation at 94 °C for 5 s and annealing/extension at 60 °C for 30 s. A melting curve analysis was conducted at 95 °C for 15 s, 60 °C for 60 s, and 95 °C for 15 s. The relative changes in cytokine mRNA expression were calculated using the 2^−ΔΔCt^ method, with *β-actin* serving as the internal control gene.

### 2.11. Statistical Analysis of Data

Statistical analysis was carried out using GraphPad Prism version 10.1.2. Group differences were assessed using one-way analysis of variance (ANOVA) with Tukey’s multiple comparison test. Statistical significance was defined as * *p* < 0.05, ** *p* < 0.01, *** *p* < 0.001, while *p* > 0.05 indicated no significant difference.

## 3. Results

### 3.1. Screening for Immunodominant B Cell, CTL and HTL Epitopes

**Figure 1:**
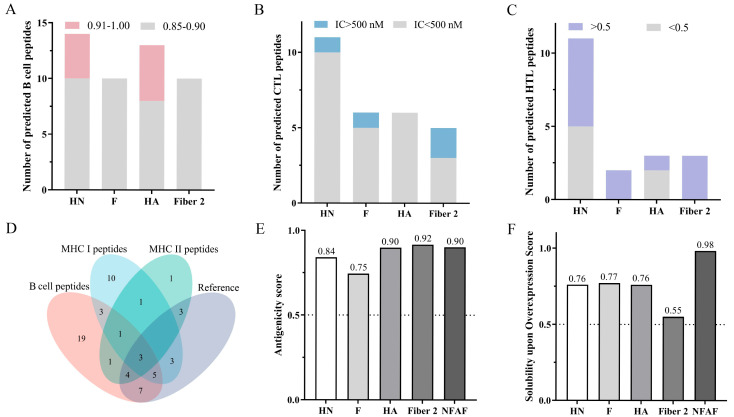
Comprehensive analysis of epitope prediction and multi-epitope vaccine evaluation. (**A**) Predicted B-cell epitopes from viral antigens using ABCpred. (**B**) CTL epitopes identified by NetMHCcons 1.1. (**C**) HTL epitopes predicted with NetMHCIIpan 4.3. (**D**) Consistency assessment between predicted and experimentally validated epitopes. (**E**) Comparative antigenicity analysis of native viral proteins versus NFAF. (**F**) Comparative analysis of solubility predictions for natural viral proteins and NFAF in *E. coli* expression System.

HN and F proteins of genotype VII NDV, HA protein of H9N2, and Fiber2 protein of FAdV-4 were selected as prediction targets based on their strong antigenic properties and demonstrated capacity to elicit neutralizing antibodies. Subsequent analysis focused on the identification of immunodominant B-cell and T-cell epitopes. Using ABCpred, a total of 38 B cell epitopes exhibiting strong binding affinity (scores ranging from 0.85 to 0.90) were predicted. Additionally, nine epitopes demonstrating extremely strong binding affinity were identified (scores ranging from 0.91 to 1.00) (Figure 1A). Detailed epitope information is summarized in Table S1. A total of 24 CTL epitopes with strong binding affinity (IC50 < 500 nM) and 4 with weak binding affinity (IC50 > 500 nM) were identified using NetMHCcons 1.1 (Figure 1B). HTL epitope analysis using NetMHCIIpan 4.3 revealed 7 epitopes with strong immunogenic potential (percentile rank < 0.5) and 12 with weak immunogenic potential (percentile rank > 0.5) (Figure 1C). Detailed information on both CTL and HTL epitopes is provided in Table S2. Notably, 25 of the predicted epitopes matched experimentally validated epitopes from prior studies (Figure 1D), with the relevant data summarized in Table S3, thereby supporting the high reliability of the prediction results. Candidate epitopes were prioritized based on their multifunctional nature, encompassing B-cell, CTL, and HTL epitopes, and supported by literature evidence. Ultimately, three epitopes were selected from genotype VII NDV HN protein, two from F protein, four from H9N2 HA protein, and four from FAdV-4 Fiber2 protein.

### 3.2. Construction and Evaluation of Multi-Epitope Vaccines

Based on the hydrophilicity index analysis of candidate epitopes obtained from ExPASy-ProtParam, the epitopes were arranged in the order HN-F-HA-Fiber2 and connected using the linker GPGPGLRMKLPKS, with hydrophilicity gradually increasing from the center toward the periphery. This design led to the construction of a 469 amino acid recombinant protein named NFAF. NFAF exhibited an antigenicity score of 0.90, comparable to that of the four original proteins (Figure 1E), suggesting strong immunogenic potential. Moreover, NFAF demonstrated a solubility score of 0.98 in *E. coli*, significantly higher than that of the original proteins (Figure 1F), indicating its excellent potential for soluble expression in the *E. coli* system and superior solubility compared to native proteins. Importantly, NFAF was confirmed to be non-allergenic and non-toxic.

**Figure 2:**
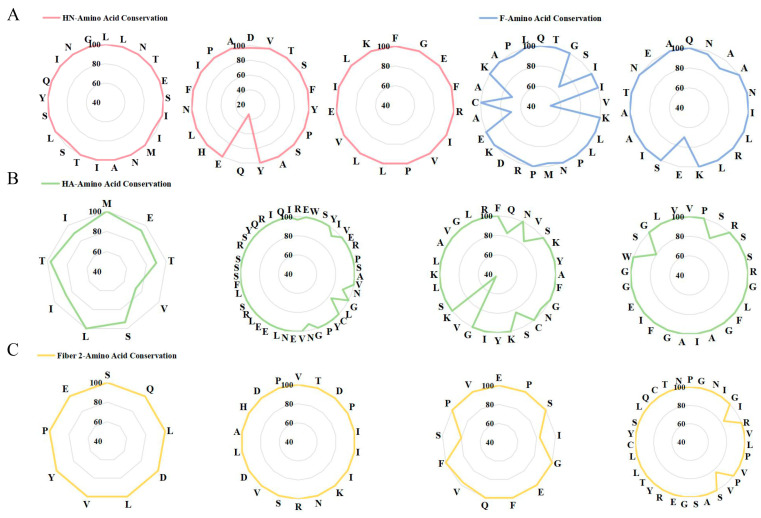
Conservation analysis of candidate antigenic epitopes across different (**A**) genotype VII NDV strains, (**B**) H9N2 strains, and (**C**) FAdV-4 strains.

To evaluate the conservation of the selected 13 epitopes, multiple sequence alignment was performed on the amino acid sequences of 90 genotype VII NDV, H9N2, and FAdV-4 strains. The results revealed 8 epitopes with 80~100% conservation levels, contrasted by 3 epitopes where 1~2 specific amino acid positions exhibited 30~70% conservation rates (Figure 2). These findings suggest that the majority of the selected epitopes are highly conserved across different viral strains, supporting their potential for broad-spectrum immunogenicity.

### 3.3. Secondary/Tertiary Structure Prediction and 3D Model Refinement

**Figure 3:**
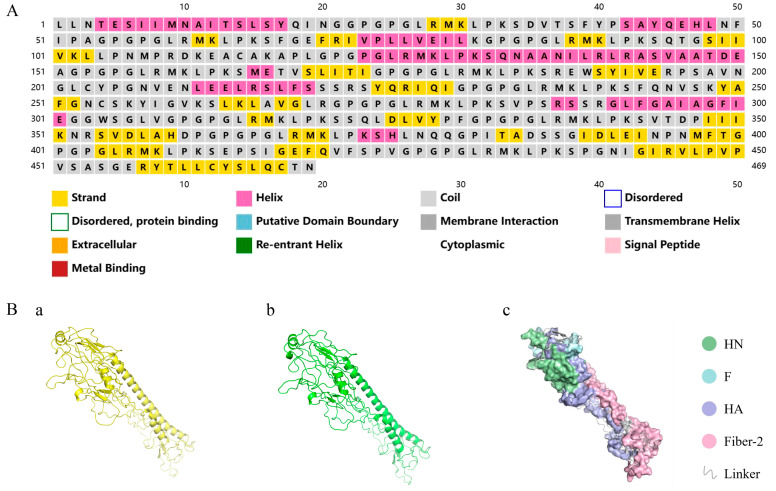
Secondary and tertiary structures of NFAF. (**A**) NFAF sequences and secondary structure through PSIPRED server. (**Ba**) NFAF tertiary structures constructed by I-TASSER. (**Bb**) Tertiary structure optimization of NFAF by Galaxy Refine server. (**Bc**) 3D structural analysis of NFAF.

The secondary structure of NFAF was analyzed through the PSIPRED server. The prediction results indicated that the protein’s structural elements consisted of 55.5% coils, 23.9% α-helices, and 20.6% β-sheets (Figure 3A). In order to confirm the correct presentation of the epitope and the stability of its structure, five 3D structural models of NFAF were generated by I-TASSER, and the model with the highest C-score (Figure 3(Ba)) was selected for further refinement. NFAF structure was subsequently refined using GalaxyRefine to obtain an improved tertiary structure (Figure 3(Bb)), in which the Rama favored ratio increased to 81.8%, the proportion of unfavorable rotamers was significantly reduced to 1.3%, and the MolProbity score improved from 3.707 to 2.774. These enhancements collectively indicate that the optimized model demonstrates superior stereochemical quality and conformational stability. The PyMOL visualization of NFAF (Figure 3(Bc)) revealed accurate spatial folding and well-organized sequential arrangement, with clearly delineated structural domains showing strong integration.

### 3.4. Molecular Docking of NFAF with TLRs, MHC I, and MHC II

Protein–protein docking has been widely recognized as a powerful computational approach for predicting interaction patterns between molecules and vaccine constructs. Molecular docking analyses were conducted to investigate the interactions between the designed multi-epitope vaccine NFAF and key immune receptors, specifically TLR2, TLR4, MHC I, and MHC II molecules.

**Figure 4:**
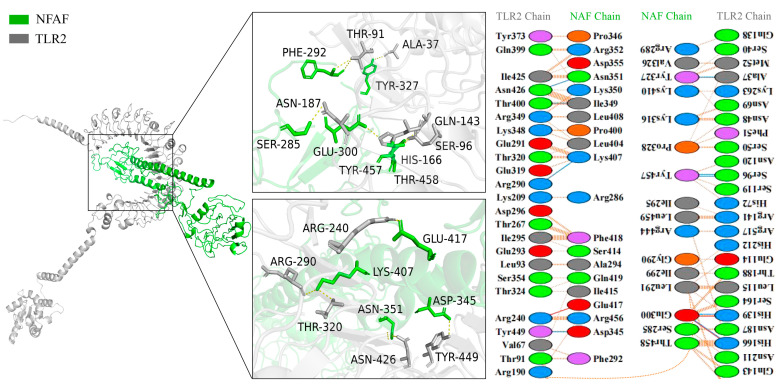
Diagram of the docking model of NFAF-TLR2 complex.

The binding interface between NFAF and TLR2 was found to consist of 11 hydrophilic amino acid residues of TLR2 (ALA37, THR-91, SER96, GLN143, HIS166, ASN187, ARG240, ARG290, THR320, ASN426, TYR449) forming a dense hydrogen bond network with 10 corresponding residues in NFAF (SER285, PHE292, GLU300, TYR327, ASP345, ASN351, LYS407, GLU417, TYR457, THR458). A 2D interaction map was generated by PDBSum, indicating that a total of 36 NFAF residues were involved in interactions with 47 residues along the TLR2 molecular chain (Figure 4).

**Figure 5:**
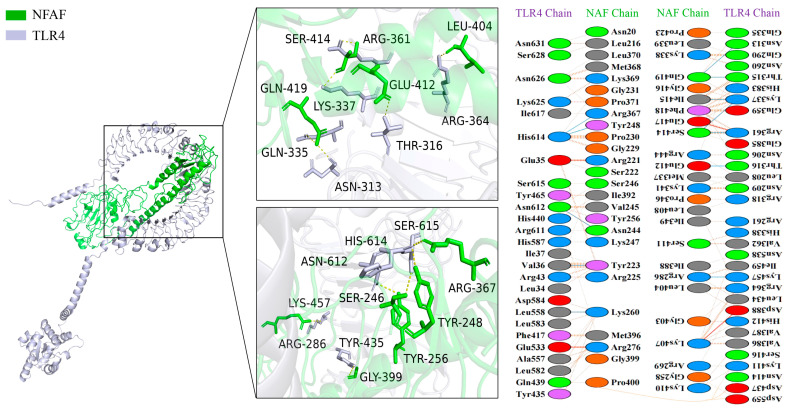
Diagram of the docking model of NFAF-TLR4 complex.

**Figure 6:**
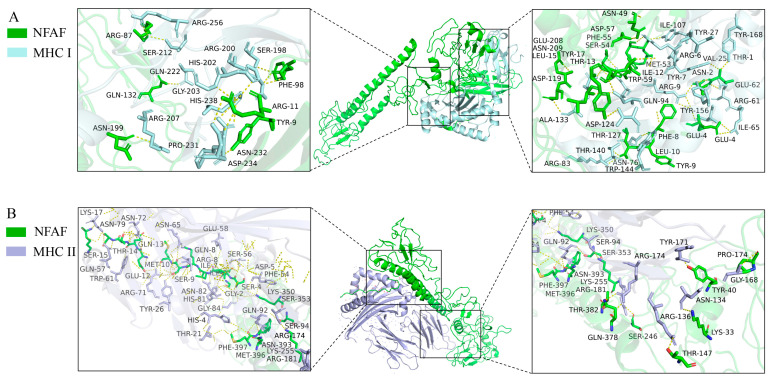
Diagram of the docking model of NFAF-MHC I and MHC II complex. (**A**) NFAF-MHC I complex; (**B**) NFAF-MHC II complex.

Similarly, more extensive interactions were observed between NFAF and TLR4, involving 12 hydrogen bonds, with 51 NFAF residues found to interact with 58 TLR4 residues (Figure 5). Notably, particularly strong interactions were demonstrated between NFAF and both MHC I and MHC II. These interactions were attributed to the inclusion of CTL and HTL epitopes in the vaccine design, providing preliminary validation of the accuracy of prior epitope prediction (Figure 6). Overall, favorable binding affinity of NFAF toward chicken TLRs and MHC molecules was demonstrated by the molecular docking results.

### 3.5. Expression and Purification of Recombinant Chimeric Protein NFAF

**Figure 7:**
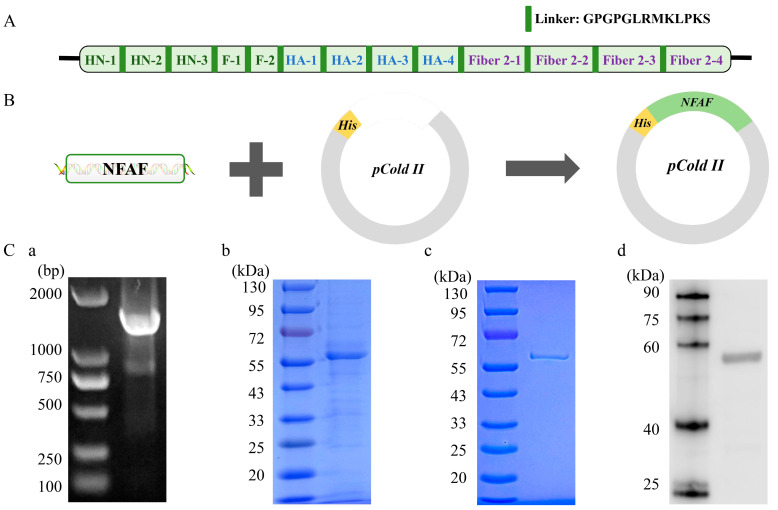
Cloning and expression of NFAF. (**A**,**B**) Cloning of NFAF in pCold II. The 13 epitopes were fused together in proper order by the appropriate linkers. (**Ca**) Agarose gel showing NFAF was amplified by the universal primers of pCold II. (**Cb**) SDS-PAGE showed NFAF expression. (**Cc**) SDS-PAGE showed NFAF purification. (**Cd**) The reactivity of anti-his tag antibody with NFAF was tested in Western blot.

The 469 amino acid NFAF protein was successfully produced through a systematic approach. A codon-optimized NFAF sequence was first connected to a flexible linker (Figure 7A) before being inserted into the pCold-His expression vector (Figure 7B). Sequence verification demonstrated perfect alignment between the constructed pCold-NFAF plasmid and the designed sequence, with no detectable deletions or mutations. Following transformation into *E. coli* BL21(DE3) competent cells (Figure 7(Ca)), protein expression was induced with 0.3 M IPTG at 18 °C for 18 h. SDS-PAGE analysis of the soluble fraction (Figure 7(Cb)) showed a prominent band at the predicted molecular weight of 56.8 kDa, confirming soluble expression of the target protein. The recombinant protein was then purified using Ni-agarose affinity chromatography, yielding a single, intense band on SDS-PAGE (Figure 7(Cc)). Specific detection of the His-tagged protein was achieved by Western blot analysis with an anti-His monoclonal antibody (Figure 7(Cd)). These results confirm the successful production of highly pure NFAF protein, which exhibits ideal characteristics for subunit vaccine development.

### 3.6. In Vitro Validation of the Antigenicity of NFAF

**Figure 8:**
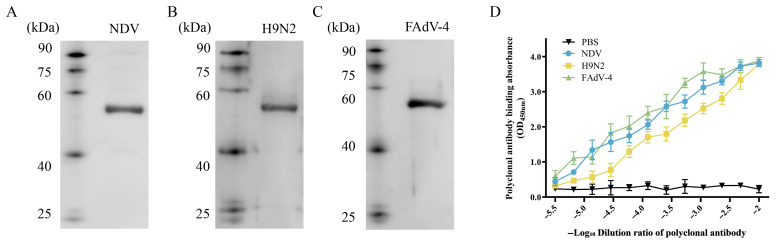
The reactogenicity of NFAF. (**A**–**C**) The reactivity of NFAF with rabbit-derived polyclonal antibodies against genotype VII NDV, H9N2, and FAdV-4 was assessed by Western blot. (**D**) The binding kinetics between NFAF and polyclonal antibodies were evaluated using an ELISA-based assay.

The key evaluation indicators for the development of protein antigenic vaccines are essential. To identify the antigenic characteristics of NFAF, Western blot and ELISA methods were used to systematically evaluate its reaction properties with genotype VII NDV, H9N2, and FAdV-4 rabbit-derived polyclonal antibodies. The Western blot results showed that at an antibody dilution of 1:200, all three virus-specific polyclonal antibodies could specifically bind to NFAF, presenting a clear 56.8 kDa characteristic band (Figure 8A–C). Further ELISA experiments analyzed the binding kinetics characteristics of NFAF with virus-specific polyclonal antibodies (Figure 8D). The results indicated that NFAF bound to the polyclonal antibodies in a dose-dependent manner and had strong antigen-binding capacity. In conclusion, these data prove that NFAF, as a tandem recombinant protein of genotype VII NDV, H9N2, and FAdV-4 epitopes, has good antigenicity and broad-spectrum immunogenicity potential.

### 3.7. Effects of NFAF on the Transcription of Cytokines in Chicken PBMC

**Figure 9:**
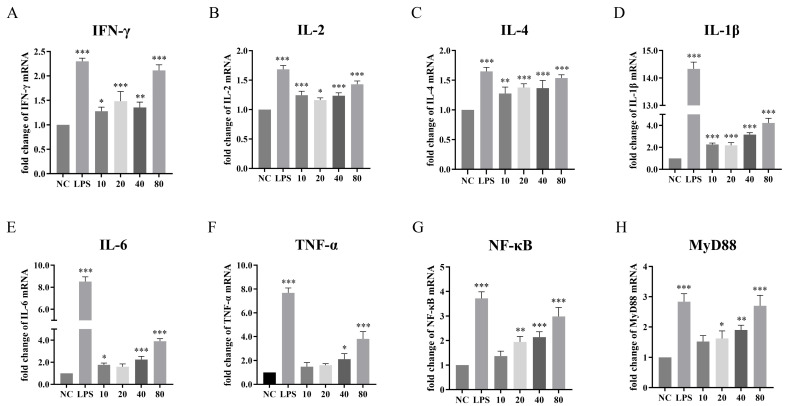
Effects of NFAF on the transcription of (**A**) *IFN-γ*, (**B**) *IL-2*, (**C**) *IL-4*, (**D**) *IL-1β*, (**E**) *IL-6*, (**F**) *TNF-α*, (**G**) *NF-κB* and (**H**) *MyD88* in chicken PBMC. The differences between groups were analyzed using Tukey’s multiple comparison of one-way ANOVA. The data was expressed as the means ± standard error of the mean (* *p* < 0.05, ** *p* < 0.01, *** *p* < 0.001).

Given that the multi-epitope vaccine NFAF integrates B-cell, CTL and HTL epitopes, its immune activation characteristics need to be verified through a systematic assessment of Th1/Th2 type immune responses. Therefore, qPCR was used to determine the influence of NFAF on the relative transcriptional levels of six important immune-related cytokines (Figure 9A–F). The results showed that NFAF stimulation enhanced the expression of these cytokines. Specifically, the expression of Th1 cytokines *IFN-γ* and *IL-2* showed a certain degree of increase, especially in the 80 μg dose group (*p* < 0.05), indicating that the vaccine can effectively activate CTL and Th1 immune responses. The moderate upregulation of *IL-4* suggests that the Th2 response is also regulated (*p* < 0.05). Notably, pro-inflammatory factors *IL-1β*, *IL-6* and *TNF-α* showed significant upregulation in all dose groups, especially in the high-dose group (*p* < 0.05), indicating that the vaccine can effectively stimulate innate immune responses. To further elucidate the molecular mechanism by which NFAF induces immune activation, the mRNA expression levels of *NF-κB* and *MyD88*, which are the key molecules in the Toll-like receptor signaling pathway, were detected (Figure 9G,H). The results showed that the transcription of both *NF-κB* and *MyD88* was significantly upregulated by NFAF stimulation in a dose-dependent. This indicated that NFAF is likely to promote the production of pro-inflammatory cytokines by activating the *MyD88* dependent *NF-κB* signaling pathway. In summary, these data indicate that NFAF, by activating cellular and humoral immune responses, demonstrates excellent immune protection potential.

## 4. Discussion

ND, AI, and HHS are the three of the most significant infectious disease threats to global poultry production. In recent years, genotype VII NDV, H9N2, and FAdV-4 have emerged as the predominant epidemic strains in many regions. However, conventional vaccines utilizing live-attenuated or inactivated viruses are often suboptimal, due to their biosafety risks and a limited ability to elicit protection against rapidly emerging immune-evading viral variants. Consequently, creating vaccines that are both safe and broadly effective against these pathogens continues to be a pressing need within the poultry industry.

In recent years, several studies have focused on the development of epitope-based vaccines targeting NDV, H9N2, and FAdV-4. For example, immunoinformatics approaches were used to identify 48 conserved epitopes from F and HN proteins of NDV, resulting in the design of two multi-epitope vaccines exhibiting high antigenicity, non-allergenic properties, and strong binding affinity to the chicken TLR7 receptor [33]. An epitope-optimized nanoparticle vaccine derived from H9N2 HA1 protein was engineered. The vaccine demonstrated potent induction of broad-spectrum neutralizing antibodies and substantially curtailed viral replication in vivo [34]. In the case of FAdV-4, multi-epitope vaccines were designed using hexon, penton, Fiber1, and Fiber2. It conferred complete protection against challenge infection [15]. These findings establish conserved epitopes within viral structural proteins as viable targets for developing broad-spectrum vaccines. Furthermore, the strategic integration of multiple antigenic epitopes proves effective in eliciting robust immune responses that exceed those achieved by single-epitope vaccine formulations.

This study systematically combined immunoinformatics design with experimental validation to integrate the immunodominant epitopes from HN and F proteins of genotype VII NDV, HA protein of H9N2, and Fiber2 protein of FAdV-4. A multi-epitope vaccine named NFAF was developed successfully, thereby achieving the design objective of combined immunization against these three pathogens. The vaccine was engineered to be non-toxic and non-allergenic, with 61.5% of its epitopes exhibiting 80~100% conservation across 90 prevalent strains, thus laying a foundation for broad-spectrum protective efficacy. Notably, the linker peptide GPGPGLRMKLPKS maintained the native conformation of all constituent epitopes while promoting the assembly of a stable α-helical domain and significantly enhances the antigen-presenting ability.

Through molecular docking analysis, the interaction capability of NFAF with chicken TLRs, MHC I, and MHC II was demonstrated. Compared to TLR2, NFAF formed 12 hydrogen bonds involving 51 interacting residues with TLR4 and exhibited a stronger binding affinity. It was demonstrated through in vitro experiments with chicken PBMC that this robust interaction leads to the activation of the *NF-κB* signaling pathway via a *MyD88*-dependent mechanism. Notably, the binding of NFAF to both MHC I and MHC II provides the possibility for the simultaneous induction of CTL and HTL responses, potentially serving as the key mechanism underlying the upregulation of *IFN-γ* and *IL-4* in chicken PBMC. NFAF was successfully expressed as a soluble protein in the *E. coli* expression system and purified, thereby validating the computational prediction regarding the solubility of the designed vaccine. ELISA and Western blot analyses demonstrated that NFAF specifically reacted with polyclonal antibodies against the three target viruses, confirming the presence of critical neutralizing epitopes derived from the original antigens. The regulation of host immune responses involves various cytokines and chemokines secreted by immune cells. These molecules mediate the synergistic action of Th1 and Th2 immune responses, collectively contributing to the defense against pathogenic microorganisms. In vitro experiments using chicken PBMC revealed that the expression levels of Th1-type cytokines *IFN-γ* and *IL-2* were significantly upregulated, indicating that NFAF successfully induced a Th1-type immune response characterized by a CTL response. The moderate upregulation of *IL-4* implies a concomitant modulation of the Th2-type immune response. Additionally, NFAF markedly enhanced the expression of pro-inflammatory cytokines such as *IL-1β*, *TNF-α*, and *IL-6*. This finding aligns with previous studies, which have shown that computer-assisted design of virus-like particle-based multi-epitope vaccines can induce strong immunogenicity by upregulating key pro-inflammatory factors such as *TNF-α*, thereby activating both humoral and cellular immune responses. In summary, NFAF induced synergistic activation of both Th1 and Th2 immune responses, substantially enhanced innate immune reactions, promoted the secretion of major pro-inflammatory cytokines, and exhibited significant immunogenic properties, and the final immune protection effect still needs to be verified in animal models.

However, the short half-life and limited delivery efficiency are significant disadvantages of multi-epitope vaccines. To overcome these limitations, future research may focus on various optimization strategies, for example, selecting adjuvants to prolong immune stimulation, introducing targeting peptides or nanocarriers to improve delivery efficiency, developing live virus vector expression systems, and incorporating biological adjuvants to enhance immunogenicity. Systematic evaluations of the immunogenicity, immune protection efficacy, and safety of multi-epitope vaccines will be conducted in SPF chicken models in the future investigation.

## 5. Conclusions

This study addresses the limitations of traditional single-antigen vaccines and proposes a theoretically feasible and highly promising strategy. A safe and effective subunit vaccine targeting genotype VII NDV, H9N2, and FAdV-4 has been designed and preliminarily validated. These research results provide a certain theoretical basis for the subsequent optimization strategy of multi-epitope vaccines.

## Figures and Tables

**Table 1:** The GeneBank numbers of 120 selected protein sequences used in this study.

HN	F	HA	Fiber2
XNJ14979.1	AFA90008.1	WNW65192.1	WGG88700.1
ACT34867.1	ANZ52445.1	AWK91180.1	ADV35562.1
ACT34869.1	AFA89995.1	QCU81185.1	ADV35559.1
ACT34870.1	AEZ36087.1	QCU81221.1	XRL28504.1
ARJ54661.1	AEZ36086.1	QQD47992.1	XRL28492.1
ACT34871.1	XNJ14935.1	QCU81173.1	WMQ56317.1
ARE59575.1	QXI73422.1	WHZ30396.1	QNI22041.1
ACT34873.1	QPL12208.1	WHZ30390.1	ULR92343.1
ACT34874.1	QPL12196.1	WHZ30385.1	ULR92225.1
AUR53331.1	ANH61879.1	WHZ30380.1	ULR92110.1
XRL22480.1	AEZ36054.1	QMY22989.1	WKF25335.1
XRL22485.1	AFA90006.1	XQV99186.1	WKF25331.1
XRL22488.1	AFA90003.1	XQV99182.1	UVI01528.1
XRL22490.1	ALR96390.1	XQV99177.1	APA19532.1
XNJ14954.1	AFA89998.1	WNW65192.1	ANV21448.1
ACT34872.1	AEZ36084.1	WHZ30395.1	WNT44153.1
QPL12203.1	QCT09563.1	WFS86944.1	WNT44151.1
AUR53391.1	AZP53700.1	XQM63907.1	WNT44149.1
ACT34875.1	AEZ36077.1	QMY23001.1	WNT44148.1
APG57100.1	ADZ96697.1	AYW17105.1	XRL28496.1
XNJ14957.1	ADH10205.1	XLZ37515.1	ULR92030.1
XNJ14961.1	AEZ36071.1	XLT98263.1	WKF25334.1
APG57082.1	UVW56757.1	WQE73161.1	UVI01743.1
APC94011.1	AVO00786.1	WJQ15874.1	WRV65845.1
AZP53670.1	AHJ81381.1	XFZ89163.1	UVI01614.1
AZP53677.1	AYN07325.1	UUG08308.1	WEW52982.1
ANZ52446.1	XYO65859.1	WPO56664.1	UNO37669.1
AGT03836.1	QLB45620.1	UXC94516.1	QLI42824.1
ADZ96704.1	AEZ36055.1	URN67694.1	AUO29792.1
CAB69409.1	AEZ36061.1	UDE32122.1	ANV21576.1

**Table 2:** Primer sequences of chicken cytokines used for qPCR.

Name	Sequence (5′-3′)	Tm (°C)	Product Size (bp)
IFN-γ	Forward: GTAGCTGACGGTGGACCTAT	56	247
	Reverse: TTCTCAAGTCGTTCATCGGGA		
IL-2	Forward: ACCAACTGAGACCCAGGAGTG	56	172
	Reverse: TCCGGTGTGATTTAGACCCGT		
IL-4	Forward: TGTAGGGGACCTGAGATGTGA	56	197
	Reverse: AGGTTGTTATCTCCACCAGGACA		
IL-1β	Forward: GGTCAACATCGCCACCTACA	56	85
	Reverse: CATACGAGATGGAAACCAGCAA		
IL-6	Forward: CGGCTTCGACGAGGAGAAA	56	228
	Reverse: TTCAGATTGGCGAGGAGGGA		
TNF-α	Forward: TGTGGGGCGTGCAGTG	56	194
	Reverse: ATGAAGGTGGTGCAGATGGG		
MyD88	Forward: CTGGCATCTTCTGAGTAGT	60	76
	Reverse: TTCCTTATAGTTCTGGCTTCT		
NF-κB	Forward: CCACAACACAATGCGCTCTG	64	112
	Reverse: AACTCAGCGGCGTCGATG		
β-actin	Forward: GCCGAGAGAGAAATTGTGCG	56	211
	Reverse: TACCACAGGACTCCATACCCAA		

## Data Availability

The original contributions presented in this study are included in the article/Supplementary Materials. Further inquiries can be directed to the corresponding author.

## Supplementary Materials

The following supporting information can be downloaded at: https://www.mdpi.com/article/10.3390/microorganisms13122744/s1, Table S1: B-cell epitopes from HN and F proteins of genotype VII NDV, HA protein of H9N2, and Fiber-2 protein of FAdV-4 were predicted using ABCpred; Table S2: CTL epitopes were predicted using NetMHCcons 1.1, while HTL epitopes were predicted using NetMHCIIpan 4.3, for HN and F proteins of genotype VII NDV, HA protein of H9N2, and Fiber-2 protein of FAdV-4; Table S3: Candidate epitopes’ information.
