# Supplementary material for: Designing a Novel Multi-Epitope Trivalent Vaccine Against NDV, AIV and FAdV-4 Based on Immunoinformatics Approaches
Source: Microorganisms. 2025 Dec 2;13(12):2744. doi: 10.3390/microorganisms13122744 (PMC12735309; doi:10.3390/microorganisms13122744)
Supplement: Supplementary file 1 [file microorganisms-13-02744-s001.zip › Table S1.pdf]

Table S1. B-cell epitopes from HN and F proteins of genotype VII NDV, HA protein of H9N2, and Fiber-2 protein of FAdV-4 were predicted using ABCpred.

Protein name	No	Sequence	Position (aa)	Score
HN	1	GVFGTMLDDEQARLNP	486	0.94
	2	TATLHSPYTFNAFTRP	435	0.93
	3	EWIYVIYKRHNNTCPD	331	0.92
	4	TRPGSVPCQASARCPN	448	0.91
	5	TGVYTDPYPLIFHRNH	467	0.89
	6	AKSSYKPGRFGGKRVQ	355	0.89
	7	GRLGFDGQYHEKDLDT	273	0.89
	8	PDEQDYQIGMAKSSYK	345	0.88
	9	LRSINLDDTQNRKSCS	224	0.88
	10	DVTSFYPSAYQEHLNF	147	0.88
	11	ESIIMNAITSLSYQIN	100	0.88
	12	PVSAVFDNISRSRVTR	501	0.87
	13	HRNHTLRGVFGTMLDD	479	0.86
	14	YSTGASTPHDLAGIST	46	0.85
F	1	TGSIIVKLLPNMPRDK	58	0.89
	2	KGSVIAKCKITTCRCT	387	0.89
	3	QGSVSTSGGRRQKRF	103	0.89
	4	TCRCTDPPGIISQNYG	398	0.87
	5	NNSISNALDRLAESNS	471	0.86
	6	GITMWVSGEFDATYQK	431	0.86
	7	GSGIEELDTSYCIESD	327	0.85
	8	SGLITGYPILYDSQTQ	266	0.85
	9	CIRPTSSLDGRPLAAA	25	0.85
	10	ALYNLAGGNMDYLLTK	237	0.85
HA	1	YPTQDAQYTNNQGKNI	169	0.97
	2	MWGINHPPTDTAQTNL	187	0.96
	3	TCTIEGLIYGNPSCDP	72	0.93
	4	LENQKTLDEHDANVNN	440	0.93
	5	TQKNNAYPTQDAQYTN	163	0.91
	6	TETVDTLTENNVPVTH	31	0.89
	7	DDQIQDIWAYNAELLV	423	0.87
	8	YGHILSGESHGRILKT	264	0.87
	9	METIRNGTYNRRKYQE	487	0.86
	10	RALGSNAVEDGRGCFE	462	0.86
	11	DSTQKAIDKITSKVNN	377	0.85
	12	VPSRSSRGLFGAIAGF	332	0.85
	13	PSAVNGLCYPGNVENL	101	0.85
Fiber 2	1	EPSIGEFQVFSPVVTG	403	0.89
	2	SVTSPWTYSANGYYEP	389	0.89
	3	PSGIQAGTVSPSTATL	364	0.89
	4	TFVSGSPSLNTYNATT	285	0.87

5	DVKVDGVTVMVNDDWE	121	0.87
6	PVPVSASGERYTLLCY	431	0.86
7	HLNQQGPITADSSGID	172	0.86
8	GTMIVGPVLYSCPAAS	462	0.85
9	SGERYTLLCYSLQCTN	437	0.85
10	SGVGVSVDESLQIVNN	221	0.85
