# Supplementary material for: Designing a Novel Multi-Epitope Trivalent Vaccine Against NDV, AIV and FAdV-4 Based on Immunoinformatics Approaches
Source: Microorganisms. 2025 Dec 2;13(12):2744. doi: 10.3390/microorganisms13122744 (PMC12735309; doi:10.3390/microorganisms13122744)
Supplement: Supplementary file 1 [file microorganisms-13-02744-s001.zip › Table S2.pdf]

Table S2. CTL epitopes were predicted using NetMHCcons 1.1, while HTL epitopes were predicted using NetMHCIIpan 4.3, for HN and F proteins of genotype VII NDV, HA protein of H9N2, and Fiber-2 protein of FAdV-4.

Protein name	Sequence (CTL)	Position (aa)	IC50 (nM)	Sequence (HTL)	Position (aa)	Rank (%)
HN	REAKNTWRL	13	32.24	LLNTESIIMNAITSLSYQING	96	0.12
	TEDKVTSLL	64	200.68	LALGVLRTSATGRVFF	206	1.2
	KQVALESPL	84	533.84	YPLIFHRNHTLRGVF	474	0.15
	TESIIMNAI	97	79.14	AVFDNISRSRVTRVSSS	504	1.8
	QEHLNFIPA	155	131.59	TTSTCFKVVKTNKAYCLSIA	527	0.4
	TEEEDYKSI	255	487.32	SIIMNAITSLSYQIN	101	0.17
	HEKDLDTTV	280	187.05	LGVLRTSATGRVFFS	208	1.3
	DEQDYQIGM	344	271.69	DPYPLIFHRNHTLRGVFGT	472	0.4
	DEQARLNPV	492	309.35	LLMVMTLAISAAALV	31	1.2
	GEFRIVPLL	552	43.41	QQAILSIKVSTSLGK	370	1.7
	HQYLALGVL	203	392.49	RIMLILGCIRPTSSL	18	1.9
F	KEACAKAPL	73	31.21	QTGSIIVKLLPNMPRDKEA	57	0.6
	RQKRFIGAV	113	210.14	QNAANILRLRASVAATDEA	146	0.9
	AQITAAAAL	133	246.48			
	LEALSVSTT	303	294.65			
	EELDTSYCI	331	131.59			
	RELDCIKIT	195	725.49			
HA	METVSLITI	1	59.09	NCSKYIGVKSLKLAVGLR	313	0.3
	FQNVSKYAF	303	288.34	ELRSLFSSSRSYQRIQI	118	1.5
	IEGGWSGLV	348	456.69	FGNCSKYIGVKSLKLA	311	0.3
	SEVETRLNM	409	105.99			
	LERQKIEGV	506	425.67			
	LESEETYKI	516	135.94			
Fiber 2	SQLDLVYPF	36	165.16	PGNIGIRVLPVPVSAS	422	1.2
	FEPMANRSV	382	405.44	VTDPIIIKNRSVDLAHDP	76	0.7
	GEFQVFSPV	407	31.88	NIGIRVLPVPVSASG	424	1.7
	QQWNIQGLL	315	797.69			
	LEINPNMFT	188	979.25			
