# Supplementary material for: Designing a Novel Multi-Epitope Trivalent Vaccine Against NDV, AIV and FAdV-4 Based on Immunoinformatics Approaches
Source: Microorganisms. 2025 Dec 2;13(12):2744. doi: 10.3390/microorganisms13122744 (PMC12735309; doi:10.3390/microorganisms13122744)
Supplement: Supplementary file 1 [file microorganisms-13-02744-s001.zip › Table S3.pdf]

Table S3. Candidate epitopes information.

Protein name	Position (aa)	Epitope sequence	Hydropathicity	B	MHC I	MHC II	Reference
	15	REAKNTWRL	-1.767		√		√
	30	LLMVMTLAISAAALVYS	2.088			√	
	46	YSTGASTPHDLAGIST	-0.188	√			
	66	TEDKVTSLL	-0.144		√		√
	86	KQVALESPL	0.033		√		√
	96	LLNTESIIMNAITSLSYQING	0.481	√	√	√	√
	123	CGAPVHDPDYIGGIGK	-0.169	√			√
	147	DVTSFYPSAYQEHLNFIPA	-0.189	√	√		√
	203	HQYLALGVLRTSATGRVFFSTLRSINLDDTQNRKSCS	-0.308	√	√	√	
	257	TEEEDYKSI	-1.800	√			
HN	273	GRLGFDGQYHEKDLDTTV	-1.002	√	√		
	305	GSFIDDRVWFPVYVGL	0.600	√			
	331	EWIYVIYKRHNNTCPDEQDYQIGM	-1.042	√	√		√
	355	AKSSYKPGRFGGKRVQQAILSIKVSTSLGK	-0.443	√		√	√
	392	PNTITLMGAEGRILTV	0.531	√			
	435	TATLHSPYTFNAFTRPGSVPCQASARCPN	-0.352	√			
	467	TGVYTDPYPLIFHRNHTLRGVFGTMLDD	-0.264	√		√	
	486	GVFGTMLDDEQARLNPV	-0.153	√	√		
	501	PVSAVFDNISRSRVTRVSSS	-0.085	√		√	√
	527	TTSTCFKVVKTNKAYCLSIA	0.360			√	√
	553	FGEFRIVPLLVEILK	1.133	√		√	√
	25	CIRPTSSLDGRPLAAA	0.100	√			
	58	QTGSIIVKLLPNMPRDKEACAKAPL	-0.100	√	√	√	√
F	103	QGSVSTSGGRRQKRF	-1.447	√			
	113	RQKRFIGAV	-0.389		√		
	133	AQITAAAAL	1.456		√		

	146-164	QNAANILRLKESIAATNEA	-0.047			√	√
	195	RELDCIKIT	-0.089		√		
	237	ALYNLAGGNMDYLLTK	0.138	√			
	266	SGLITGYPILYDSQTQ	-0.119	√			
	303	LEALSVSTT	0.789		√		
	327	GSGIEELDTSYCIESD	-0.463	√			
	331	EELDTSYCI	-0.278		√		
HA	1	METVSLITI	1.467		√		
	31	TETVDTLTENNVPVTH	-0.588	√			√
	53	TEHNGMLCA	-0.144		√		
	72	TCTIEGLIYGNPSCDP	-0.013	√			√
	92	REWSYIVERPSAVNGLCYPGNVENLEELRSLFSSSRSYQRIQ	-0.547	√	√	√	√
	163	TQKNNAYPTQDAQYTNNQGKNI	-1.882	√			√
	187	MWGINHPPTDTAQTNL	-0.738	√			
	208	TTTSVATEEMNRIFKP	-0.556	√			
	264	YGHILSGESHGRILKT	-0.406	√			
	303	FQNVSKYAFGNCSKYIGVKSLKLAVGLR	0.121		√	√	
	332	VPSRSSRGLFGAIAGFIEGGWSGLV	0.544	√	√		√
	377	DSTQKAIDKITSKVNN	-1.075	√			
	409	SEVETRLNM	-0.733		√		
	423	DDQIQDIWAYNAELLV	-0.144	√			
	440	LENQKTLDEHDANVNN	-1.606	√			√
	462	RALGSNAVEDGRGCFE	-0.506	√			√
	487	METIRNGTYNRRKYQE	-2.056	√			
	506	LERQKIEGVKLESEETYKILTI	-0.545		√		
Fiber 2	36	SQLDLVYPF	0.433		√		
	76	VTDPIIIKNRSVDLAHDP	-0.156			√	√
	121	DVKVDGVTVMVNDDWE	-0.250	√			

172	HLNQQGPITADSSGIDLEINPNMFT	-0.424	√	√		
221	SGVGVSVDESLQIVNN	0.275	√			√
285	TFVSGSPSLNTYNATT	-0.181	√			
315	QQWNIQGLL	-0.356		√		
364	PSGIQAGTVSPSTATL	0.256	√			
382	FEPMANRSVTSPWTYSANGYYEP	-0.861	√	√		√
403	EPSIGEFQVFSPV	0.215	√	√		√
422	PGNIGIRVLPVPVSASGERYTLLCYSLQCTN	0.265	√		√	√
462	GTMIVGPVLYSCPAAS	1.069	√			
